# Glutathione Peroxidase of *Pennisetum glaucum* (PgGPx) Is a Functional Cd^2+^ Dependent Peroxiredoxin that Enhances Tolerance against Salinity and Drought Stress

**DOI:** 10.1371/journal.pone.0143344

**Published:** 2015-11-23

**Authors:** Tahmina Islam, Mrinalini Manna, Malireddy K. Reddy

**Affiliations:** 1 Plant Molecular Biology Group, International Centre for Genetic Engineering and Biotechnology, Aruna Asaf Ali Marg, New Delhi, 110067, India; 2 Department of Botany, University of Dhaka, Dhaka, 1000, Bangladesh; National Taiwan University, TAIWAN

## Abstract

Reactive oxygen species (ROS) arise in the plant system due to inevitable influence of various environmental stimuli. Glutathione peroxidases are one of the important ROS scavengers inside the cell. A glutathione peroxidase (*PgGPx*) gene was previously found from *Pennisetum glauccum* abiotic stressed cDNA library. Enzyme kinetics data revealed that PgGPx possessed preference towards thioredoxin rather than glutathione as electron donor and thus belongs to the functional peroxiredoxin group. Moreover, its activity was found to be dependent on divalent cations, especially Cd^2+^ and homology model showed the presence of Cd^2+^ binding site in the protein. Site directed mutagenesis study of PgGPx protein revealed the vital role of two conserved Cysteine residues for its enzymatic activity and structural folding. Expression analysis suggested that *PgGPx* transcript is highly up-regulated in response to salinity and drought stresses. When expressed ectopically, *PgGPx* showed enhanced tolerance against multiple abiotic stresses in prokaryotic *E*. *coli* and model plant, rice. Transgenic rice plants showed lesser accumulation of MDA and H_2_O_2_; and higher accumulation of proline as compared to wild type (WT) plants in response to both salinity and drought stresses that clearly indicates suppression of lipid peroxidation and ROS generation in transgenic lines. Moreover, transgenic plants maintained better photosynthesis efficiency and higher level of antioxidant enzyme activity as compared to WT plants under stress conditions. These results clearly indicate the imperative role of PgGPx in cellular redox homeostasis under stress conditions, leading to the maintenance of membrane integrity and increased tolerance towards oxidative stress.

## Introduction

Glutathione peroxidases (GPxs) are non haem-thiol peroxidases that belong to a pervasive anti-oxidant enzyme family. It catalyses the reduction of hydrogen peroxide (H_2_O_2_) to water and other lipid hydroperoxides to corresponding alcohols with the use of reduced glutathione (GSH). Due to its broad spectrum of substrate specificity and higher affinity towards H_2_O_2_; GPxs have been subject of numerous studies related to oxidative stresses. It has been found to be highly important in the detoxification of stress induced reactive oxygen species and thus protect cells from serious oxidative damages [1].

Most of the animal GPx enzymes differ from its plant counterpart because of the presence of a Seleno-Cys (SeCys) residue in their catalytic active site in place of Cys [2]. Due to greater nucleophilic affinity of SeCys than Cys, animal GPx enzymes are believed to be catalytically more active. However, substitution of Cys with SeCys in the active site of plant GPx was unable to increase GSH-dependent peroxidase activity [3]. Unlike animal GPxs, plant GPxs showed higher preference for Thioredoxin (TRx) instead of GSH [4]. Based on the involvement of conserved Cys residues in reaction mechanism, plant peroxiredoxins (Prxs) could be divided into four subgroups; such as 2-cys Prx, 1-cys Prx, type II Prx and Prx Q [5]. During peroxide reduction, first a highly conserved Cys residue is transformed into sulfenic acid and regenerated back into reduced form by various ways based on the type of Prxs. Among them, 2-cys Prxs reduces sulfenic acid with two Cys by forming an intermolecular disulfide bond whereas Prx Q class do the same with an intramolecular disulfide bond [5]. In general, disulfide bonds are again reduced back to their normal state by reduction of Trx. In other two subgroups, there is no disulfide bond formation due to the lack of second conserved Cys residue [6]. Plant type II Prxs could be reduced back by either glutaredoxin (Grx) or Trx-system or both [7], whereas only Trx-system was able to reduce 1-Cys Prxs [8]. On the basis of biochemical evidence, Rouhier and Jacquot (2005) [9] suggested that plant GPxs constituted the fifth group of large thioredoxin-dependent peroxidase family (Prxs).

Plant GPxs have been reported to be involved in various biotic and abiotic stress adaptation pathways. Increased accumulation of *GPx* transcripts have been shown in different plant species in response to exogenous hormone treatments [10], salinity stress [11], heavy metal toxicity [12], oxidative damage [13] and programmed cell death [14]. Apart from abiotic stresses, expression of *OsPHGPx* has been found to be highly up-regulated in response to pathogen infection [15] and physical wounding [16]. Plant GPxs have been reported to perform multiple functions such as cell cycle regulation [17], H_2_O_2_ scavenging, signal transduction, redox sensing etc [18]. Recently, crystal structure of Poplar GPx was identified that possess 32 Cd^2+^ ion binding sites in a single asymmetric unit. Thus GPxs are proposed to act as a Cd^2+^-sink during Cd^2+^ toxicity [2]. Considering this broad spectrum eminence of GPxs, it would be notable to characterize a GPx gene from naturally stress tolerant plant, *Pennisetum glaucum* (namely *PgGPx*).

Thus, it is very relevant to characterize PgGPx in terms of its expression pattern, enzyme kinetics, reaction mechanism, and stress tolerance potential. Here, we have performed expression analysis of *PgGPx* transcript, and detailed enzyme kinetic analysis including reaction condition optimization, metal dependency, and structural folding. We show that PgGPx is a Cd^2+^ dependent functional peroxiredoxin. Moreover, heterologous expression of PgGPx in *E*. *coli* and model plant rice provides enhanced tolerance against salinity and drought stresses.

## Materials and Methods

### Plant Growth Conditions, Stress Treatment and qRT PCR

Pearl millet (*Pennisetum glaucum* (L) R. Br) seeds were surface sterilized with 1% Bavistin for 20 min and grown on vermiculite under greenhouse conditions (14/10 h light/dark cycle illumination at 370 μEm^-2^s^-1^ and 30± 2°C). Two weeks old seedlings were subjected to salinity stress (200 mM NaCl) or drought stress (withdrawal of water) for 48 h. Plants irrigated with water served as control. Rice (*Oryza sativa* L ssp. japonica) seeds were grown in controlled environmental condition of 28±2°C temperature and 16 h photoperiod in growth chamber. Seeds were surface sterilized for 20 min with 1% Bavistin and allowed to germinate in a hydroponics system supplemented with Yoshida medium [19]. Rice seedlings of 10 d age were chosen for various stress treatments. RNA was isolated and cDNA was prepared according to manufacturer’s protocol (Thermo Scientific, USA). qRT PCR was performed as previously described [20].

### Purification of Recombinant PgGPx and Enzyme Kinetics

*E*. *coli* BL21 (DE3), harbouring the pET28a_*PgGPx* plasmid was grown in liquid LB medium till OD_600_ reached about 0.5 and was subsequently induced with 1 mM isopropyl *β*-D-1-thiogalactopyranoside (IPTG) for 16 h at 18°C. Recombinant PgGPx protein was purified by Ni-NTA affinity chromatography and confirmed by western blotting using anti-His antibodies as described previously [21]. Glutathione peroxidase assay for the purified recombinant protein or total plant protein extracts was performed according to Weydert and Cullen (2010) [22] with slight modification. The molar absorption coefficient of NADH at 340 nm is 6,220 M^−1^cm^−1^ and was used to calculate the specific activity of GPx. Effect of various metal ions (such as Cd^2+^, Zn^2+^, Co^2+^, Fe^2+^, Cu^2+^, Mn^2+^) on PgGPx activity was tested by adding them individually in the assay buffer. To chelate metal ions, PgGPx protein was incubated with 10 mM EDTA overnight at 4°C followed by overnight dialysis against 10 mM MOPS, pH 7.2. The specific activity of the enzyme was expressed in l mol min^-1^ mg^-1^ of protein. Enzyme kinetic parameters of PgGPx were determined by measuring activity at varying concentration of substrate and electron donor. Different enzyme kinetics parameters were calculated by extrapolating Hanes–Woolf plot.

### Homology Modeling of PgGPx

A homology based structural model of PgGPx was built using SWISS-MODEL program (http://swissmodel.expasy.org) based on PDB structure (2p5q) as template. Modelled structure was verified using NIH server (http://nihserver.mbi.ucla.edu/SAVES/). Ramachandran plot calculated from NIH server was used to validate the predicted model. All the images were generated using chimera (http://www.cgl.ucsf.edu/chimera) [23].

### Site-Directed Mutagenesis of PgGPx, Expression and Purification of the Muteins

The site directed mutagenesis was carried out according to the manufacturers protocol (Thermo Scientific Phusion Site-Directed Mutagenesis Kit) using 5´-phosphorylated PAGE purified primers and pET28a_*PgGPx* plasmid as template. After confirming by sequencing, muteins were expressed and purified as described above for wild type (WT) protein.

### Assessment of *E*. *coli* Growth Pattern

BL21 (DE3) *E*. *coli* cells transformed either with empty vector pET28a alone or pET28a_*PgGPx* were grown in 5 ml LB media at 37°C (A_600 ~_ 0.45). After induction of the culture by 1 mM IPTG, cells were exposed to different abiotic stress inducing agents (such as NaCl for salinity stress or mannitol for drought stress). A growth curve was made by taking OD_600_ of the bacterial culture after every 2 h interval till it reached the stationary phase.

### Generation and Confirmation of Rice Transgenic Plants

An efficient rice transformation protocol [24] was used with minor modifications for the development of transgenic plants. Southern hybridization of putative transgenic plants was carried out using DIG non-radioactive nucleic acid labelling and detection system (Roche, Germany). Expression of *PgGPx* was checked by semi quantitative RT-PCR [21] followed by peroxidase assay [22].

### Leaf Strip Senescence and ROS Staining

Leaves were collected from both WT and transgenic plants of equal age and height. Then leaf strips were floated on water (as experimental control) or 200 mM NaCl (to impose salinity stress) or 150 mM mannitol (to impose drought stress) [25]. The leaf strips were observed for 72 h, followed by spectrophotometric measurement of total chlorophyll content. Cellular accumulation of H_2_O_2_ and O_2_^-^ was visualized histochemically according to previous studies [21, 26]. All the measurements were repeated three times, taking three individual replicates each time.

### Assessing Biochemical and Physiological Parameters of Transgenic Plants

Two week old rice seedlings were exposed to salinity (200 mM NaCl) or drought stress (150 mM mannitol) for 24 h. Electrolytic leakage measurement was performed as previously described by Sairam *et al*., 2005 [27]. Measurement of proline, MDA and H_2_O_2_ was performed from the WT and transgenic seedlings according to Turan and Tripathy, 2013 [28]. Antioxidant enzymes activity was carried out according to Lata *et al*., 2011 [29]. All experimental data were recorded as the means of three independent experiments.

### Measurement of Photosynthetic Parameters

Different components of photosynthetic machinery such as rate of photosynthesis, rate of transpiration, stomatal conductance and chlorophyll fluorescence (F_v_/F_m_) were determined in the third to fifth expanded leaves using an infra-red gas analyzer (Li-COR 6400–40, Lincoln, USA) with default settings. All these measurements were repeated three times with three replicates each time.

## Results

### Isolation, Cloning and Sequence Analysis of *PgGPx*

Full length *PgGPx* gene was amplified by RACE-PCR using stress responsive *Pennisetum glaucum* EST clone [30] as template and specific primers (S1 Table). *PgGPx* consists of an ORF of 507 bp that encodes a protein of 168 amino acids with an apparent molecular weight of 18.6 kDa and pI of 5.48. The deduced amino acid sequence of PgGPx showed distinguishable homology with other reported GPxs from diverse species such as rice, wheat, tomato, citrus, *Arabidopsis*, bacteria and yeast etc. Multiple sequence alignment analysis showed the presence of conserved GPx domain and GLUTPROXDASE fingerprint in PgGPx (S1 Fig).

### *PgGPx* Showed Upregulation in Response to Salinity and Drought Stresses

**Fig 1:**
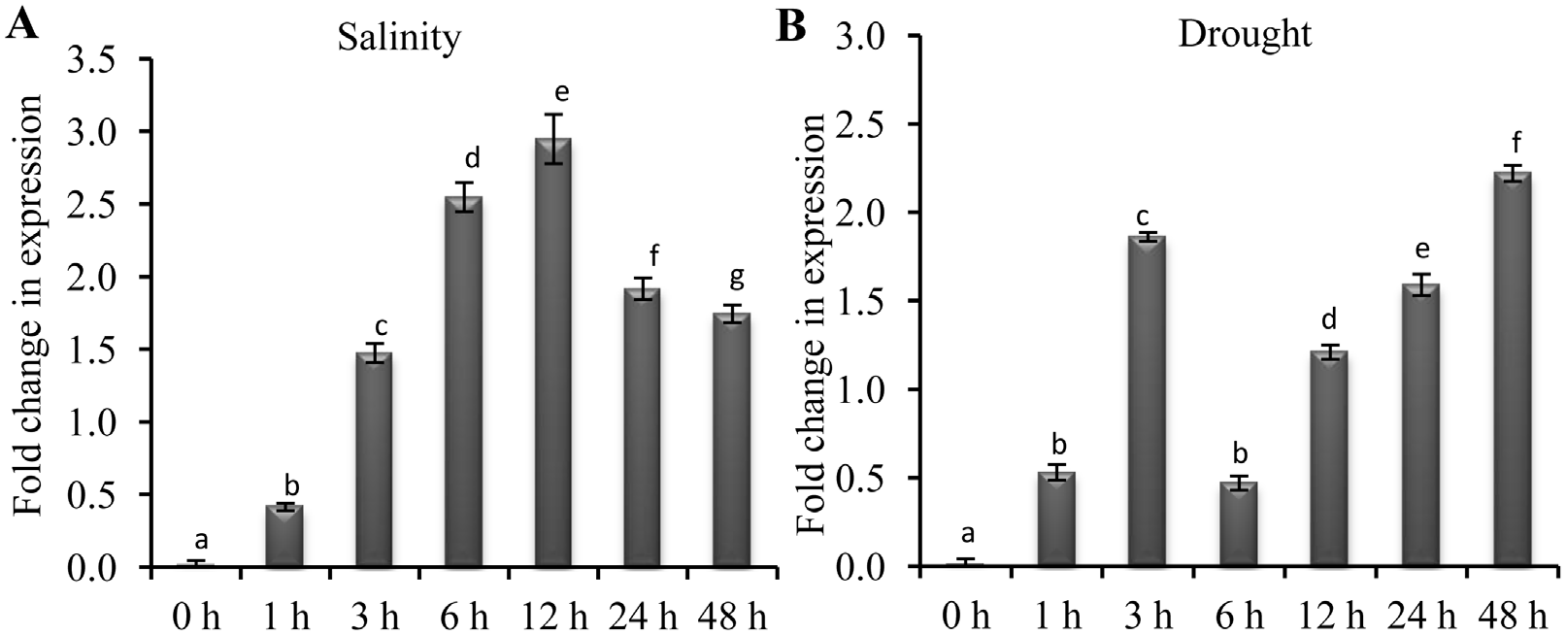
Stress responsive expression analysis of *PgGPx*. Alteration of *PgGPx* transcripts were determined by qRT-PCR analysis after exposing *Pennisetum gluccum* seedlings with 200 mM NaCl for salinity stress or 150 mM manitol for drought stress for 48 h. Histograms depicts the fold change in expression of *PgGPx* under salinity (A) and drought (B) stresses. Steady-state mRNA level of *PgGPx* was normalized with respect to house keeping gene *tubulin* and fold change in expression were calculated as compared to control sample (0 h). Values are presented as mean ± standard deviation of three independent experiments. Statistical significance was determined using two-tailed paired Student’s t-test and are represented with different letters (p < 0.05).

To elucidate the role of PgGPx under salinity and drought stresses, a time dependent expression analysis of *PgGPx* transcript was performed (Fig 1). A significant upregulation (1.5 fold) of *PgGPx* was observed as early as 3 h after salinity treatment. After that, a gradual increase was observed till 12 h (approximately 3-fold; Fig 1A), followed by a maintenance of 1.5 fold up-regulation till 48 h. However, a steady up-regulation of *PgGPx* transcript was observed till 48 h of drought stress, except 3 h time point (Fig 1B). Expression profile of *PgGPx* clearly demonstrated the imperative and prolonged role of PgGPx towards salinity and drought stresses adaptation pathways.

### PgGPx Shows Thioredoxin Dependent Peroxidase Activity

**Fig 2:**
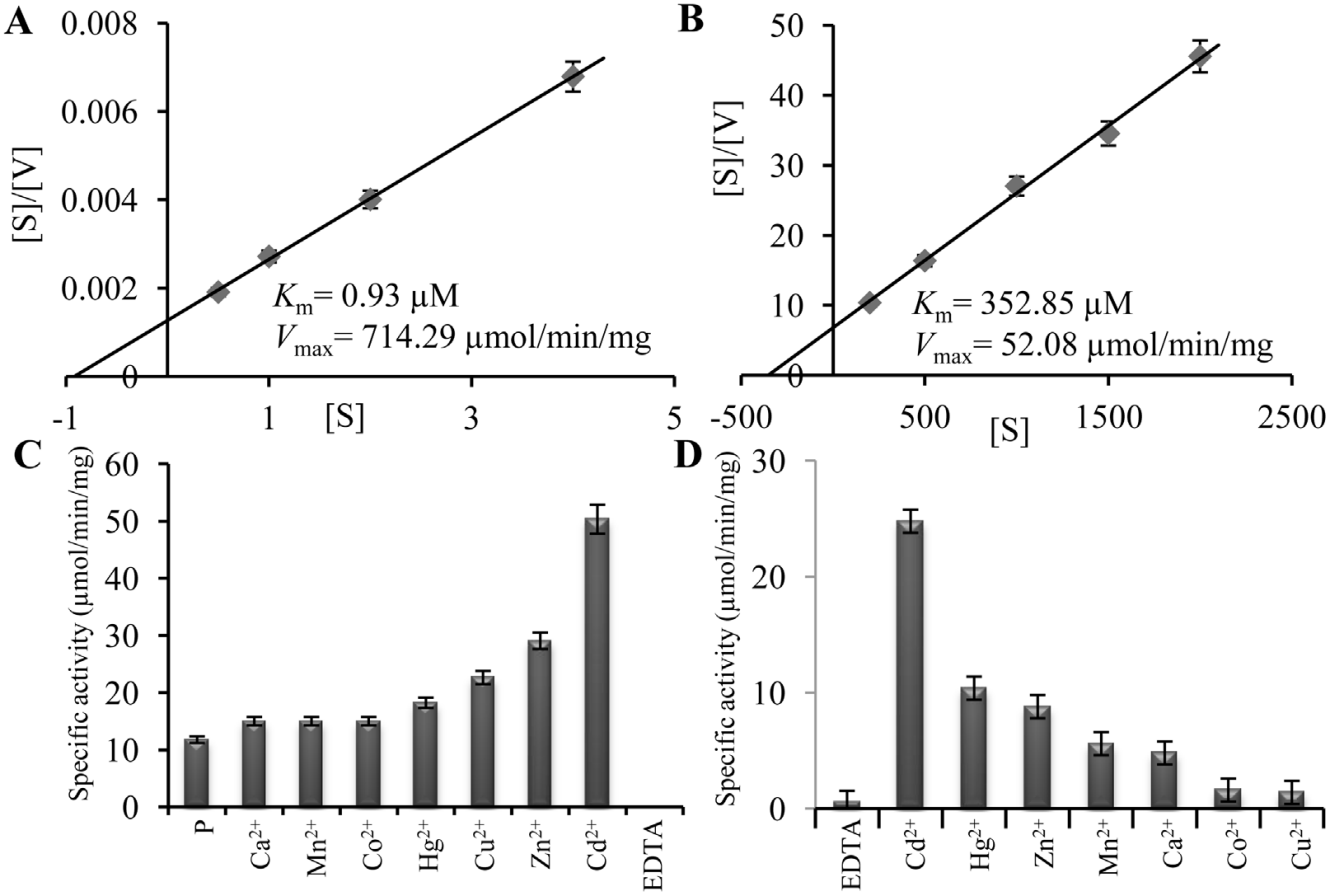
PgGPx illustrates thioredoxin dependent peroxidase activity and reliant on Cd^2+^. Enzyme kinetics of PgGPx was performed in presence of Trx (A) or GSH (B) as electron donor and different parameters were calculated. Hanes-Woolf plot was generated to depict the enzyme kinetics of PgGPx in the presence of thioredoxin and thioredoxin reductase (A); or glutathione and glutathione reductase (B). Enzyme kinetics values were shown in the inset of each figure. (C) PgGPx activity was measured in presence of various metal ions, as well as without metal addition as purified (P) and ethylene diamine tetra acetic acid (EDTA) treated form. Activity was immensely increased by the addition of Cd^2+^, however it was completely abolished in the EDTA chelated form. (D) Relative reactivation of EDTA treated PgGPx was analyzed by the addition of various exogenous divalent metal ions. Note that the maximum specific activity was achieved with Cd^2+^. Experiments were repeated three times with three replicates each time.

To determine the enzymatic activity of PgGPx, the corresponding cDNA was cloned into a bacterial expression vector pET28a (S2A Fig) and recombinant PgGPx protein was purified to homogeneity by Ni-NTA based affinity chromatography. Protein was visualized on 12% SDS polyacrylamide gel as a single 20 kDa protein (S2B Fig). Glutathione peroxidase activity of the recombinant PgGPx was assayed using tert butyl hydroperoxide (t-BHP) and Thioredoxin (Trx) as a substrate. A typical bell-shaped curve was observed with a maximal activity at pH 7.2 and 37°C of temperature when assayed using a wide range of pH (6.0–10.0) and temperatures (0°C to 60°C) (S3A and S3B Fig). Glutathione peroxidase could use three different electron donors, such as GSH, NADPH and Trx. Thus, enzyme kinetics of PgGPx was measured with three different electron donars, separately. PgGPx showed activity in presence of Trx and GSH (Fig 2), except NADPH (data not shown). Enzyme kinetic analysis showed that PgGPx had higher substrate affinity (low *K*_m_) and apparent activity (*V*_max_) towards Trx by 350 and 14 times, respectively than GSH (Fig 2A and 2B). Thus, PgGPx functions as a thioredoxin-dependent peroxidase (TPx).

### PgGPx Requires Divalent Cations for Its Optimum Activity

Peroxidase activity of PgGPx was verified in the presence of various cations (Cd^2+^, Zn^2+^, Ca^2+^, Mn^2+^, Cu^2+^, Hg^2+^ and Co^2+^) and 10 mM EDTA (metal chelating agent) to check the effect of metal on its activity. Most of these divalent cations enhanced PgGPx activity, whereas EDTA resulted in complete loss of activity (Fig 2C). Among all the cations, Cd^2+^ showed the highest potential to increase PgGPx activity. Metal dependency of PgGPx was further proved by measuring the reactivation of metal chelated PgGPx protein in presence of various metals. It was observed that, exogenous addition of various divalent cations (such as Cd^2+^, Hg^2+^, Zn^2+^, Mn^2+^, Ca^2+^, Co^2+^, Cu^2+^) were able to reactivate PgGPx activity at various degrees (Fig 2D). A significant restoration of PgGPx activity was observed after reconstitution with Cd^2+^. Interestingly, Cd^2+^ reconstituted PgGPx protein showed higher activity than purified non-EDTA treated PgGPx protein (Fig 2C, p). This indicated that all the active sites of purified recombinant PgGPx protein might not be saturated with essential Cd^2+^ and complete saturation with excess Cd^2+^ ion showed maximum activity (Fig 2C, Cd^2+^).

### Structural Model of PgGPx Confers Presence of Cd^2+^ in Its Active Site

**Fig 3:**
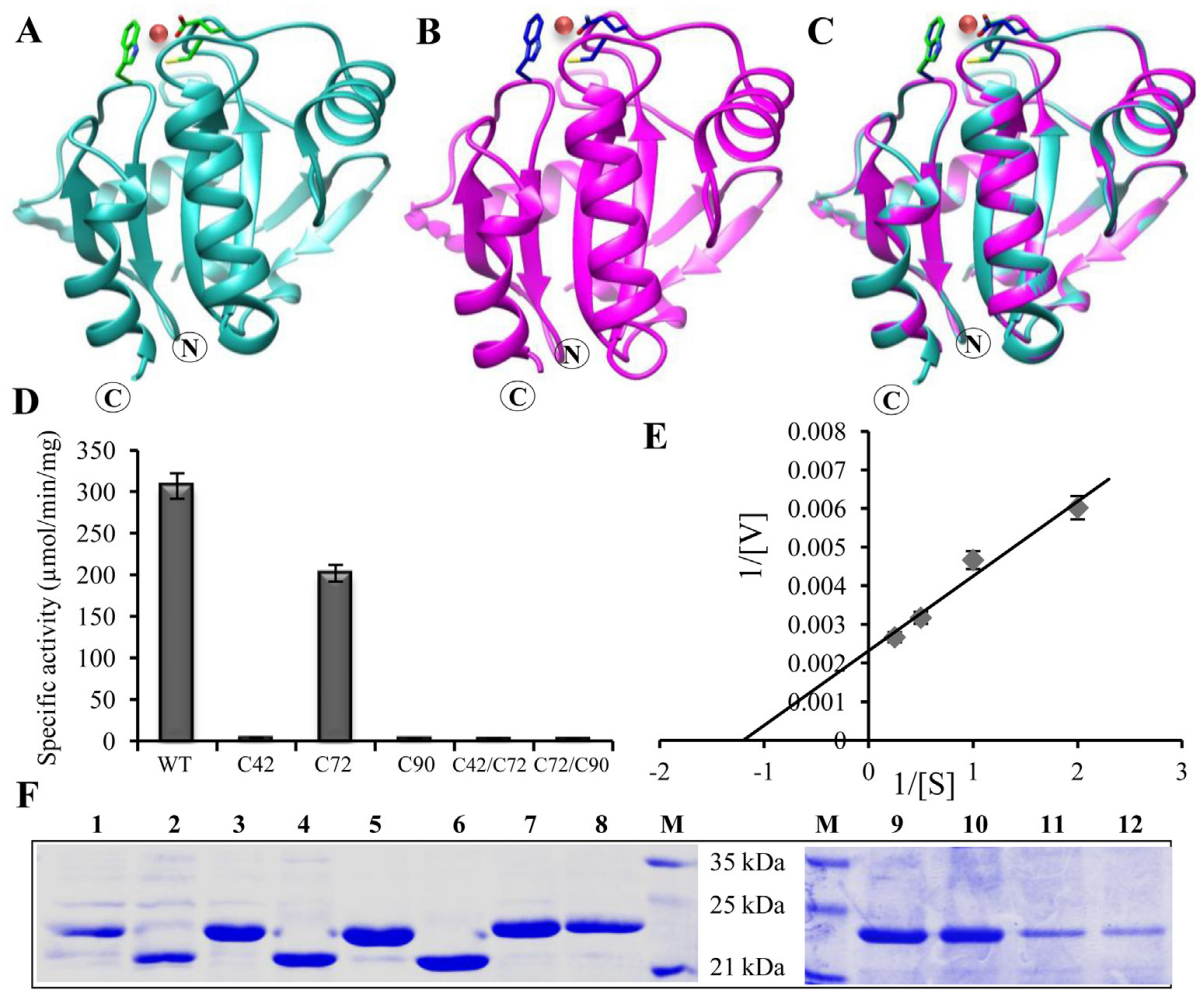
Cys residues are decisive for the thioredoxin dependent PgGPx activity. Homology model of PgGPx (B) was built based on Poplar GPx structure (2p5q) as template (A). (C) Superimposed model of both the structures onto each other. PgGPx active site possess a cadmium ion (pink color ball), coordinated to the peroxidatic Cys 42, Trp 131 and Glu 77 residues. (D) Specific activities were calculated using WT and all other muteins in response to thioredoxin dependent system. Note that all the muteins become almost inactive except C72. (E) Lineweaver-Burk plot was generated to determine different enzyme kinetic parameters for C72 protein. (F) Effects of redox potential on the conformation of PgGPx. Wild type (WT) proteins along with all muteins were either oxidized (lanes 1, 3, 5, 7, 9, and 11) or reduced (lanes 2, 4, 6, 8, 10, and 12) with 30 mM H_2_O_2_ or 30 mM DTT, respectively and separated on 14% SDS- PAGE.

Previous studies have indicated the presence of bound Cd^2+^ to active site of Poplar GPx [2]. To know the coordination of metal with active site residues, a homology-based structure of PgGPx was built (Fig 3B) based on the available crystal structure of Poplar glutathione peroxidase 5 (PDB ID: 2p5q) (Fig 3A). According to the model, PgGPx is a monomeric protein consisting of four α-helical loops and seven β-sheets (Fig 3B). In case of the arrangement of α-helices, three of them α1, α2 and α4 were found to be located on the same side and α3 was situated on the other side. The central β-core sheet was formed by the anti-parallel combination of β3, β4, β5, and β6. Based on this β core sheet, two of the helices α1 and α4 were found to be parallel and resided onto the central β strands. The other two α helices (α2 and α3) were oriented perpendicular to each other. The N-terminal end of the protein exhibited two extra chains that folded into the β-sheet. Superimposition of both template and modelled structures (Fig 3C) indicated that PgGPx had similar binding site for Cd^2+^. PgGPx active site possesses a cadmium ion, coordinated to the peroxidatic Cys 42, Trp 131 and Glu 77 residues. This might be one of the possible reasons behind the Cd^2+^ dependent PgGPx activity (Fig 2C).

### PgGPx Is a Functional 2-Cys-Peroxiredoxin

Conserved Cys residues of GPx proteins play significant role both in enzyme catalysis and structural arrangement. So all three Cys residues of PgGPx were mutated and a total of seven variants of PgGPx (C42S, C72S, C90S, C42/72S, C42/90S, C72/90S and C42/72/90S) were generated by replacing Cys with Ser. In terms of enzyme kinetics, all muteins except C72S showed complete loss of peroxidase activity (Fig 3D). Although C72S showed similar affinity for substrate (*K*_m_), enzyme turnover rate and reaction velocity decreased drastically as compared to native WT PgGPx (Fig 3E). Thus conserved Cys residues of 42 and 90, except 72 are essential for Trx regeneration and enzyme catalysis.

It was reported previously that the activity of GPx was dependent on the formation of redox-dependent disulphide bonds [6]. We have examined the redox state of WT PgGPx and its Cys/Ser variants by performing denatured polyacrylamide gel electrophoresis under different conditions. In reducing conditions (30 mM DTT), all the muteins including WT migrated at their expected molecular mass, around 20 kDa (Fig 3F; Lanes 1, 3, 5, 7, 9 and 11). Under oxidizing conditions (30 mM H_2_O_2_), WT PgGPx along with its C42S and C72S variants shifted to an apparent lower molecular mass (around 16 kDa) (Fig 3F; Lanes 2, 4 and 6). But the migration profiles of C90S, C42/72S and C72/90S muteins were not altered (Fig 3F; Lanes 8, 10 and 12). Alteration in migration profile for all the proteins in which Cys90 was not mutated indicates the possibility to form an intra-molecular disulphide bond involving Cys 90 under oxidizing conditions. Cys 90 might form disulfide bond with either Cys 72 or Cys 42 in case of C42S or C72S muteins, respectively. These observations indicate that Cys42 and Cys90 form an intra-molecular disulfide bridge. Thus PgGPx could be considered as functional 2-cys peroxiredoxin.

### Ectopic Expression of *PgGPx* Confers Multiple Stress Tolerance to *E*. *coli* and Rice

Stress tolerance potential of *PgGPx* was initially evaluated in a prokaryotic system *E*. *coli* (BL21) cells. It was found that *E*. *coli* cells transformed with pET28a_*PgGPx* had enhanced survival ability as compared to empty pET28a vector transformed cells during salinity and drought stress conditions in a concentration-dependent manner (S4 Fig). Ability of *PgGPx* to confer stress tolerance in *E*. *coli*, suggested its crucial role in stress resistance and prompted us to validate its protective role in plant system.

**Fig 4:**
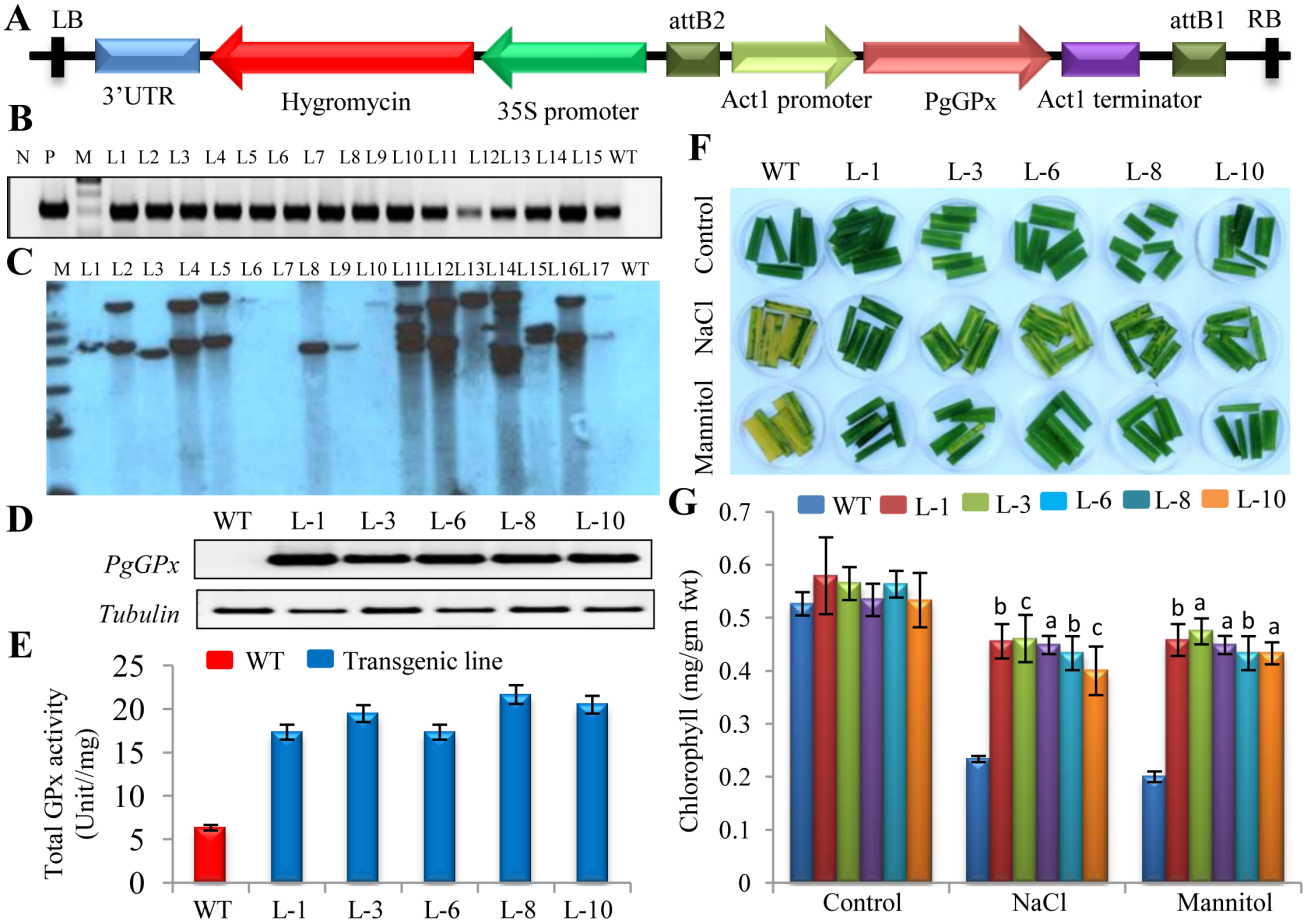
Confirmation of the ectopically expressed *PgGPx* transgenic rice plants and their stress tolerance potential. (A) Schematic illustration of the pMDC99-PgGPx expression vector used to overexpress *PgGPx* in rice plants. Putative transgenic lines were initially screened by PCR using *PgGPx* gene specific primers (B) and further confirmed by Southern blot analysis using full length *PgGPx* cDNA as probe (C). To evaluate the expression of *PgGPx* transcript in the 14 days old WT and five single copy T_1_ transgenic lines (L-1, L-3, L-6, L-8 and L-10) under normal condition, Reverse transcription PCR (RT-PCR) was carried out (D) and compared with house keeping gene, *Tubulin*. (E) Total glutathione peroxidase activity was determined from WT and transgenic plants (L-1, L-3, L-6, L-8 and L-10). Rapid leaf strips senescence assay of transgenic vis-a-vis WT plants was performed, to assess the stress tolerance under 200 mM NaCl or 150 mM mannitol. Leaf strips floated on water served as the experimental control (G). Total chlorophyll contents were estimated from the corresponding control, salt and mannitol treated leaf strips of WT as well as transgenic lines. The data represent means±standard deviation (STD) of three biological replicates (n = 3). Statistically significant differences were determined using two-tailed paired Student’s t-test as compared with WT plants of similar conditions and indicated by ^a^,P < 0.001 or ^b^,P < 0.01 or ^c^,P < 0.05.

For this, *PgGPx* was cloned into pMDC99 plant transformation vector under the control of constitutive Act1 promoter (Fig 4A) and rice transgenic plants were raised by utilizing *Agrobacterium*-mediated transformation protocol [24]. A considerable higher transformation efficiency of 14% was observed using this transformation protocol (S2 Table). Putative transgenic lines were initially screened by PCR using *PgGPx* gene specific primers (Fig 4B), and eventually confirmed by Southern blot hybridization (Fig 4C). Semi-quantitative RT-PCR of single copy transgenic lines (L-3, L-8 and L-10) was performed to examine the *PgGPx* mRNA expression level (Fig 4D). Expression analysis of *PgGPx* transcript by RT-PCR showed the presence of *PgGPx* transcript in all the transgenic plants, except WT. But all plants showed clear expected size band in case of housekeeping gene *Tubulin* (endogenous control) that was used to check the quality of cDNA (Fig 4D). Total GPx activity was measured from total protein extracts of WT and transgenic plants. As compared to WT plants, transgenic lines showed significantly higher peroxidase activity (Fig 4E).

Stress tolerance potential of *PgGPx* overexpressing lines was examined preliminary by a rapid leaf strips senescence assay under salinity and drought stress conditions. Leaf strips of transgenic lines as well as WT plants were collected from the plants of same age and approximately equal in height, and floated on water (experimental control) or 200 mM of NaCl (for salinity stress) or 150 mM of mannitol (for drought stress). Significant differences were observed in their level of chlorophyll retention within 24 h, and monitored till 72h (Fig 4F), followed by chlorophyll estimation (Fig 4G). The level of chlorophyll loss was about 15 to 20% in the transgenic lines, as compared to more than 50% in the WT line in response to salinity and drought (Fig 4G).

### Ectopic Expression of PgGPx Enhanced the Phenotypic Morphogenesis and Resist Accumulation of ROS under Stress

**Fig 5:**
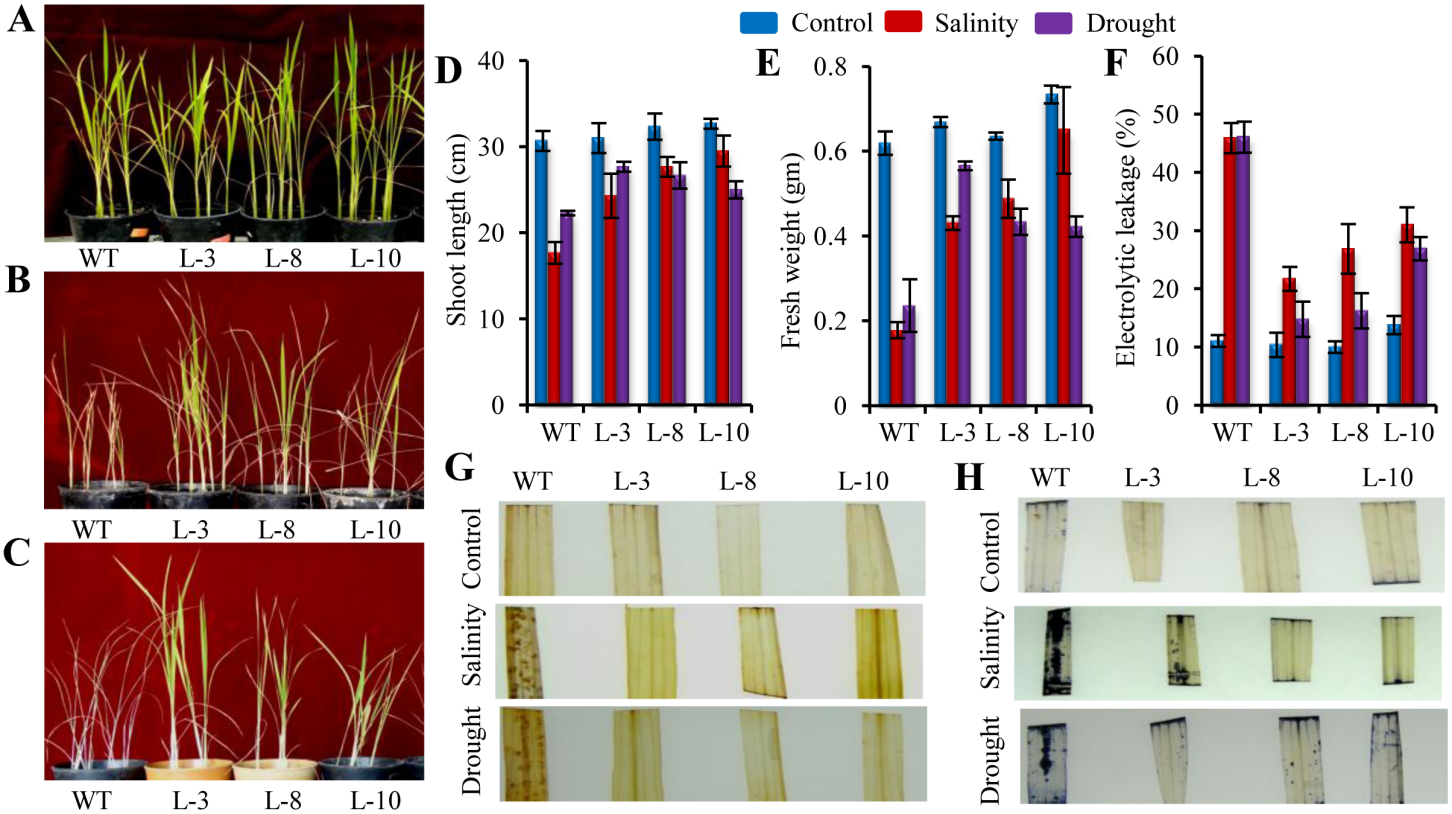
Assessment of salinity and drought tolerance of *PgGPx* overexpressing T_1_ transgenic seedlings. Growth of the transgenic as well as WT rice plants grown under control condition (A) or in the presence 200 mM NaCl (B) or 150 mM mannitol (C). Comparison of shoot length (D) and fresh weight (E) of transgenic vis-a-vis WT plants under salinity and drought stresses showed that transgenic plants had better physiological growth. Analysis of cellular damage in response to salinity and drought were measured by electrolytic leakage (F). Relative H_2_O_2_ and O^2-^ accumulation in the transgenic and WT plants under salinity and drought stresses were assessed by histochemical staining. Total H_2_O_2_ and O^2-^ levels were visualized by 3,3 0-diaminobenzidine (DAB) staining (G) and nitro blue tetrazolium (NBT) staining (H), respectively.

Seedlings from WT and transgenic lines displayed contrasting growth pattern under control and stress conditions (Fig 5A–5C). Total fresh weight and shoot length of the seedlings were drastically reduced in the WT plants in comparison to the *PgGPx* transformed transgenic plants under both salinity and drought stresses (Fig 5D and 5E). Stress induced membrane damage affected WT seedlings to lose significant amount of electrolytes in comparison to transgenic lines (Fig 5F). Lower rate of electrolytes leakage indicate better membrane stability of transgenic lines as compared to WT. Moreover, transgenic plants showed better resistance towards the accumulation of reactive oxygen species (H_2_O_2_, O_2_^-^) in contrast to WT lines, during both salinity and drought stresses (Fig 5G and 5H). Accumulations of H_2_O_2_ and O_2_^-^ were visualized by 3,3’-diaminobenzidine (DAB) staining and nitro blue tetrazolium (NBT) staining, respectively. In experimental control condition, there was no significant variation between the transgenic and WT lines in terms of H_2_O_2_ and superoxide O_2_^-^ staining. However, their levels increased significantly in the WT line in response to salinity and drought stresses in contrast to transgenic lines (Fig 5G and 5H).

### Transgenic Plants Showed Less ROS Accumulation and Lipid Peroxidation as Compared to WT under Stresses

**Fig 6:**
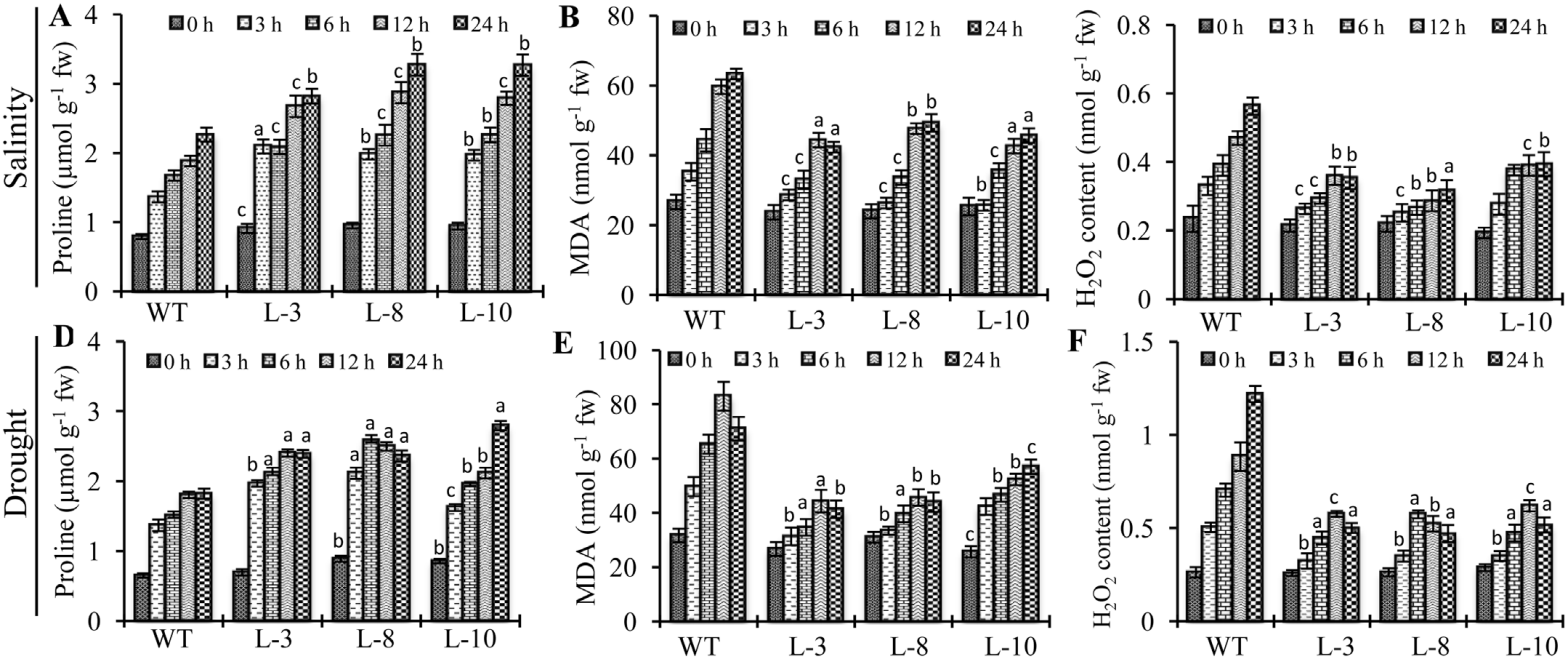
Biochemical analysis of T1 *PgGPx* transgenic lines under salinity and drought stresses. Different biochemical parameters such as proline, MDA and H_2_O_2_ was determined in transgenic vis-a- vis WT plants, under salinity (A-C) and drought (D-F) stresses at different time points (0 to 24 hr.). Alterations in the level of proline accumulation (A, D), lipid peroxidation expressed in terms of Malondialdehyde (MDA) content (B, E), and hydrogen peroxide (H_2_O_2_) content (C, F) were measured from the the transgenic and WT plants. Data represent the average of three replicates ± Standard deviation. Statistically significant differences were determined using two-tailed paired Student’s t-test as compared with WT plants of similar conditions and indicated by ^a^,P < 0.001 or ^b^,P < 0.01 or ^c^,P < 0.05.

Modulations of ROS machinery enticed by salinity and drought stresses were authenticated by the measurement of malondialdehyde (MDA), proline and H_2_O_2_ content in transgenic vis-a-vis WT plants (Fig 6). Transgenic plants showed three to four folds higher accumulation of proline in response to both salinity (Fig 6A) and drought (Fig 6D) stresses whereas only two fold upregulation was observed in WT as compared to their respective controls (0 hr value). On the other hand, MDA accumulation was significantly higher in WT as compared to transgenic plants under both stresses (Fig 6B and 6E). This indicates higher degree of lipid peroxidation in WT plants as compared to transgenic plants under stress.

Furthermore, as GPx is one of the main members of H_2_O_2_ detoxifying mechanisms in plant, level of total H_2_O_2_ was measured from the WT and transgenic plants under salinity and drought conditions (Fig 6C and 6F). WT plants showed three folds increase in total H_2_O_2_ level after 24 h of salinity stress, whereas transgenic lines showed only 1.5 to 2 folds increment (Fig 6C). Similarly in response to 24 h of drought stress, WT plants showed six folds increase in their H_2_O_2_ content, whereas it was only two to three folds in the transgenic lines (Fig 6F). To comprehend the underlying mechanism of H_2_O_2_ homeostasis by transgenic plants, we have assayed the activities of various antioxidant enzymes i.e. superoxide dismutase (SOD), ascorbate peroxidase (APX), catalase (CAT) and glutathione reductase (GR) from transgenic and WT plants (Table 1). It was observed that transgenic plants had increased antioxidant enzyme activities under both salinity and drought stresses as compared to WT plants. Not only the increase of GPx activity, but the overall increase of the anti-oxidant pathway, ultimately leads to the lower accumulation of ROS under stress in the transgenic lines.

**Table 1:** Comparison of various anti-oxidant enzymes activity of WT and transgenic plants under control, salinity and drought conditions.

Parameters	Conditions	WT	L-3	L-8	L-10
**SOD activity (Units/mg protein)**	**Control**	2.783333	2.75	2.713333	2.843333
	**Salinity**	3.416667	4.496667	5.046667	5.11
	**Drought**	3.25	3.966667	4.3	4.323333
**APX activity (Units/mg protein)**	**Control**	3.01	3.016667	3.04	3.04
	**Salinity**	3.076667	5.073333	5.003333	5
	**Drought**	2.88	4.573333	4.253333	4.63
**CAT activity (Units/mg protein)**	**Control**	2.033333	2.096667	2.146667	2.133333
	**Salinity**	3.34	6.533333	6.526667	6.9
	**Drought**	3.166667	6.326667	6.253333	6.25
**GR activity (Units/mg protein)**	**Control**	0.219667	0.232	0.223333	0.209667
	**Salinity**	0.293333	0.406667	0.43	0.43
	**Drought**	0.28	0.383333	0.406667	0.41

### Enhanced Stress Tolerance by Transgenic Lines Could Be Directly Correlated with Their Better Photosynthesis Efficiency and Yield Production

**Fig 7:**
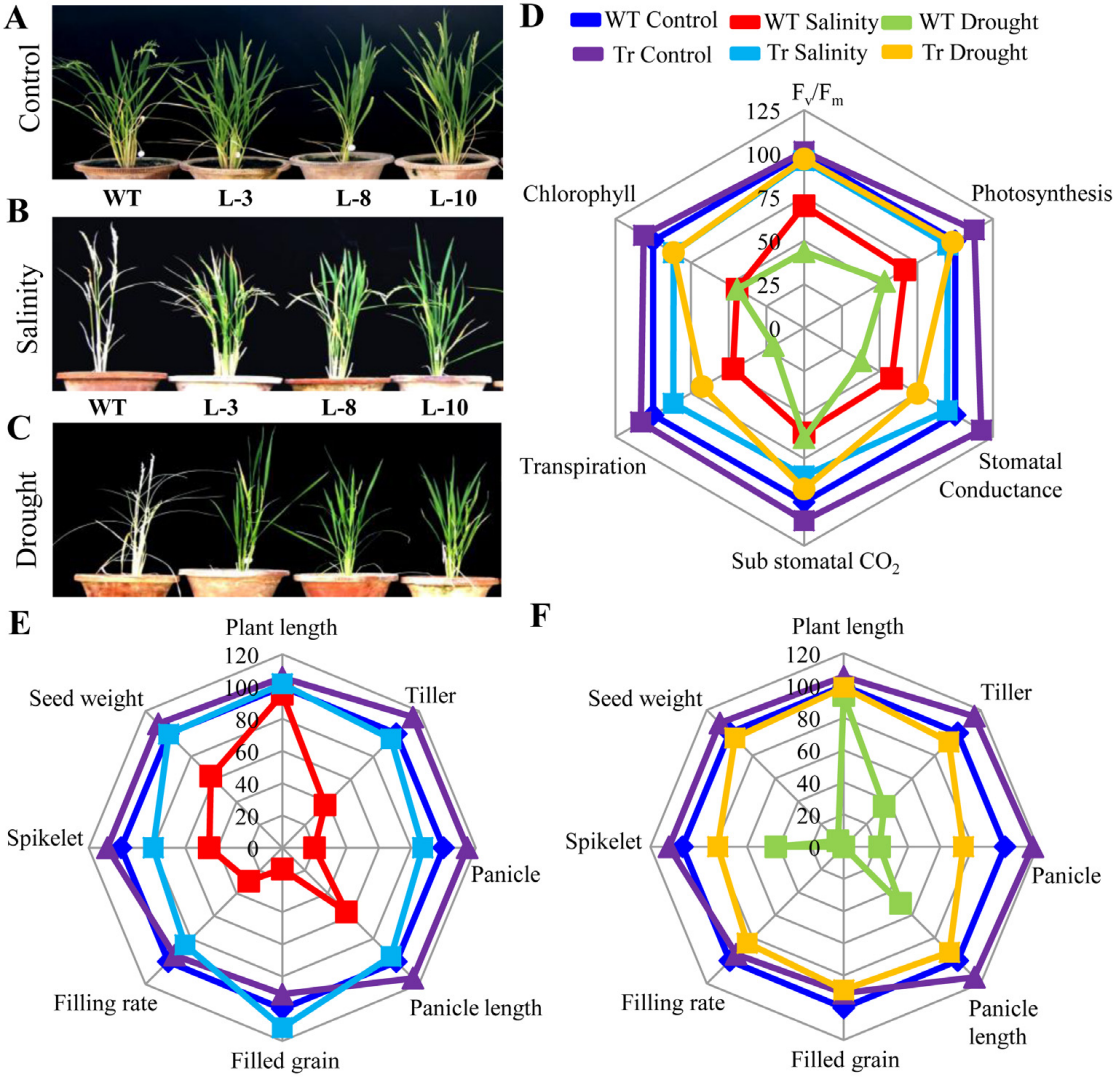
Transgenic plants ectopically expressing *PgGPx* maintained physiological balance under salinity and drought stresses. (A-C) Mature transgenic as well as WT rice plants grown in normal condition (A) or in presence 200 mM NaCl (B) or 150 mM mannitol (C). Spider plot (D) was generated by comparing various photosynthetic parameters of three independent transgenic (average of three transgenic lines) plants and corresponding WT under control, salinity and drought stresses. Mean values from WT controls were set at 100% as a reference. (E, F) Agronomic traits for *PgGPx* rice plants grown in the field under normal and stress conditions. Spider plots of agronomic traits of three independent T1 transgenic lines (average of three transgenic lines) and corresponding WT controls under salinity (E) and drought (F) conditions, respectively, were drawn using mean values from WT control as 100%.

To evaluate the effect of salinity and drought on the total yield of transgenic and WT plants, we imposed both the stresses on the one month old rice plants until maturity. WT plants were not able to survive under any of the stresses; whereas transgenic plants were able to cope up with both the stresses (Fig 7A–7C). Assorted photosynthetic parameters such as photosynthesis rate, total chlorophyll content, photosystem II (PSII) efficiency, stomatal conductance, and transpiration rate were measured (Fig 7D). Under control condition, *PgGPx* transgenic lines exhibited similar photosynthetic rate as compared to their non-transformed WT plants. Under stress conditions, transgenic plants were able to maintain all the parameters with minimum reduction, whereas WT plants showed drastic reduction. This might be one of the possible reasons of yield and biomass loss of WT plants under stress conditions.

To assess the relative yield potential, different parameters such as plant height, number of tillers, panicle number and length, spikelet number, filled grain rate, number of filled grains and average seed weight were measured from both WT and transgenic plants under control and stress conditions, and compared (Fig 7E and 7F). Analysis revealed that decrease in grain yield under stress conditions were significantly lower in the *PgGPx* over-expressing transgenic lines than WT. WT plants exhibited only 30% grain filling rate and 20% filled grain under salinity stress as compared to its control condition (Fig 7E), whereas there was no grain filling in case of 15d drought stress (Fig 7F). However, transgenic plants were able to maintain all the estimated parameters including grain filling rate and filled grain with minimum reduction and were able to produce similar amount of yield like WT plants under control condition. Thus, it could be inferred that overexpression of *PgGPx* gene might help plant to overcome the stress induced damages with minimum yield penalty.

## Discussion

Glutathione peroxidases (GPx) are a group of enzymes that regulate the levels of reactive oxygen species in cells, and protect the cells against oxidative damage. *GPx* genes have been isolated and characterized from various plant species such as *Nicotiana sylvestris* [31], *Citrus sinensis* [32], *Avena fatua* [33], *Pisum sativum* [34], *Lycopersicon esculentum* [35], *Populus trichocarpa* [36], *Arabidopsis thaliana* [37], *Triticum aestivum* [38] etc. and are found to be induced in response to various stresses. For example, expression of *AtGPx* was reported to be stimulated by exogenous salt, mannitol, heat, cold, Fe or Cu treatment [39]. Expression of *AtGPx* mRNA was also found to be induced by various plant hormones, such as salicylic acid, jasmonic acid, abscisic acid and indole acetic acid. *OsGPx* transcripts were found to be up-regulated in response to heavy metal toxicity [40] and abiotic stresses [41]. Significant up-regulation of *PgGPx* transcript was also observed in the present study in response towards salinity and drought stresses (Fig 1). But the pattern of *PgGPx* transcript accumulation was different in two different types of stress. Salinity stress induced a continuous increase of *PgGPx* transcript till 12 h of observation, whereas a steady up-regulation of *PgGPx* transcript was observed till 48 h of drought stress from 6h (Fig 1). In response to drought, a higher level of *PgGPx* accumulation was observed at very stage (3 h) that might lead to the transient decrease in *PgGPx* transcript level at the 6 h time point, followed by gradual increase till 48 h. This indicates the versatile role of plant GPx in the stress perception, signalling and adaptation pathways.

Although PgGPx showed higher activity in presence of Trx (Fig 2), it was quite low than the animal counterparts. This could be due to the presence of rare selenocysteine residue in animal GPxs which is replaced by a cysteine in plant GPx catalytic site [3]. PgGPx was found to be catalytically more efficient as compared to other GPxs from *Lycopersicon esculentum*, *Helianthus annuus*, *Nicotiana tabaccum* and *Populus triocarpa* (Table 2). Catalytic mechanism and structural arrangement of GPx proteins are primarily determined by the presence of three conserved Cys residues in its active site (S1 Fig). PgGPx also possesses three conserved Cys residues in its active site. Among them two are crucial for its activity as mutation of them leads to complete loss of activity (Fig 3D). A characteristic gel shift was observed in muteins as compared to WT (Fig 3F), indicating the crucial role of these Cys residues in protein folding. Similar pattern of altered redox status was reported for yeast GPx2 [42] and poplar GPx [6] after mutation of Cys residues. The redox-dependent shift clearly indicates that Cys-90 of PgGPx is essential for the formation of an intra-molecular disulfide with either Cys-42 or Cys-72. This is an additional piece of evidence which provides the clue that GPx belongs to the Prx family which form non-covalent dimer. Thus, it could be concluded that PgGPx possessed 2-Cys Prx type reaction mechanism by forming an intra-molecular disulfide bridge.

**Table 2:** Comparison of enzyme kinetic parameters of recombinant GPx proteins from different species.

Species	Electron donor	App.*V*_max_ (nmol min^-1^ mg^-1^)	*K*_m_ (μM)	*V*_max_/*K*_m_	Reference
*Pennisetum glaucum*	GSH	51.02	329.9	154.6X10^-3^	Present study
	Trx	714.29	0.86	830.23	Present study
*Lycopersicon esculentum*	GSH	48.8±4.56	9300±209	5.24X10^-3^	[43]
	Trx	263.2±0.36	2.2±0.30	119.6	[43]
*Helianthus annuus*	GSH	46.7±3.89	4900±120	9.53X10^-3^	[43]
	Trx	243±0.50	1.5±0.06	162.6	[43]
*Nicotiana tabaccum*	GSH	63.80±6.6	**_**	***_***	[44]
*Populus triocarpa*	Trx	**_**	1.41	***_***	[6]

Moreover, homology model of PgGPx reveals the presence of Cd^2+^-binding in the active site similar to Poplar GPx. Metal ions function as cofactors either by changing the structural conformation of the protein or helping in the formation of active site [45]. Both metal dependence and metal reactivation studies indicate the significant role of Cd^2+^ on the overall activity of PgGPx. To the best of our knowledge, this is the first report of a Cd^2+^-dependent GPx from any higher eukaryotes. Presence of such Cd^2+^-dependent GPx opens up possibilities of existence of other Cd^2+^-dependent enzymes in other plants. Since, high concentrations of Cd^2+^ might be toxic to plants [46], presence of Cd^2+^-dependent enzyme may imply its crucial role in Cd^2+^ ion homeostasis within plant cells. In depth investigation is required to validate the role of PgGPx in Cd^2+^ ion homeostasis.

Previously, GPxs have been reported to involve in the detoxification of endogenous ROS generated through different pathways in response to various environmental stresses [47]. Overexpression of *GPx* in rice, *Arabidopsis*, tobacco and poplar enhanced tolerance against various abiotic stresses [36, 39, 48, 49]. Stress tolerance potential of PgGPx protein was initially tested in the prokaryotic *E*. *coli* system and overexpression of PgGPx was found to provide significant stress tolerance towards salinity and drought stresses (S3 Fig). The reason might be the ability of PgGPx to detoxify the stress induced ROS. Accumulation of excess ROS inside the cell is a common consequence of all the stresses exposed to plants [21]. Thus overexpression of a major antioxidant enzyme, GPx could relieve cells from the adverse effects of ROS. Transgenic rice plants ectopically expressing *PgGPx*, showed significant increase in total GPx activity as compared to WT (Fig 4E), which resulted in their greater resistance to accumulate excess ROS (Fig 5G and 5H). Accumulation of excess ROS disturbs several physiological and cellular processes like physiological growth, membrane stability, photosynthesis efficiency and electrolytic imbalances etc. Enhanced stress tolerance of plant is directly associated with cell membrane stability and integrity [50]. Transgenic plants were able to maintain better membrane stability that could be directly correlated with their ability to prevent electrolytic leakage and maintenance of better relative water content (RWC) under salinity and drought stresses as compared to WT plants. Lesser ROS accumulation could be maintained in the transgenic lines by increase in activities of antioxidant enzymes such as SOD, APX, CAT and GR (Table 1). Increased anti-oxidant enzymes also maintained the pool of reduced glutathione and ascorbate that in turn maintained the redox homeostasis of cell. Our results showing synergistic effect of antioxidant enzymes with regard to salinity and drought stress are in agreement with previously reported studies in other species during heat, cold stress [51] and salt stress [52]. Apart from less accumulation of ROS, adaptive mechanisms of transgenic plants are also associated with higher accumulation of osmolyte, proline and lower lipid peroxidation [53]. Higher proline level and low lipid peroxidation might help plants to provide stress tolerance that has been reported earlier in wheat [27].

In our present study, transgenic plants were able to maintain better shoot and root growth as well as total fresh weight as compared to WT plants. Efficiency of photosynthetic machinery could be directly correlated with the overall growth and yield of plant. Photosynthesis machinery is affected first and foremost by the stresses. Stresses force plants to reduce rate of transpiration by lowering the stomatal conductance to preserve cellular water, which in turn reduces sub-stomatal CO_2_ concentration and decrease the total photosynthesis rate. It was observed that *PgGPx* over-expressing transgenic rice plants were able to maintain all the important components of photosynthetic machinery with minimum reduction and thus, were able to have yield better with only a minimum loss under both salinity and drought stresses. Comparative study of yield related parameters revealed enhanced yield potential of *PgGPx* overexpressing lines in comparison to WT during various abiotic stresses.

Taken together, it could be concluded that ectopic expression of *PgGPx* in rice imparts enhanced tolerance against two most intimidate environmental stresses, salinity and drought. The morphological, physiological, biochemical and yield data analysis showed that *PgGPx* over-expressing transgenic lines had considerable level of tolerance as compared to WT plants. All these results validated the crucial role of *PgGPx* in imparting stress tolerance without any yield penalty via strengthening of membrane integrity and antioxidant machinery, and maintaining of photosynthetic efficiency.

## Supporting Information

S1 Fig Multiple sequence alignment of GPx proteins from various organisms including *Pennisetum*. (TIF)

S2 Fig Cloning and heterologous expression of PgGPx into bacterial expression vector pET28a. (TIFF)

S3 Fig PgGPx activity is dependent on p^H^ and temperature. (TIFF)

S4 Fig Comparison of growth pattern of *PgGPx* transformed E. coli cells under stress. (TIFF)

S1 Table Primers used in the study. (PDF)

S2 Table Transformation efficiency of japonica rice cultivars. (PDF)
