# Supplementary figures and images for: Glutathione Peroxidase of Pennisetum glaucum (PgGPx) Is a Functional Cd2+ Dependent Peroxiredoxin that Enhances Tolerance against Salinity and Drought Stress
Source: PLoS One. 2015 Nov 23;10(11):e0143344. doi: 10.1371/journal.pone.0143344 (PMC4658160; doi:10.1371/journal.pone.0143344)

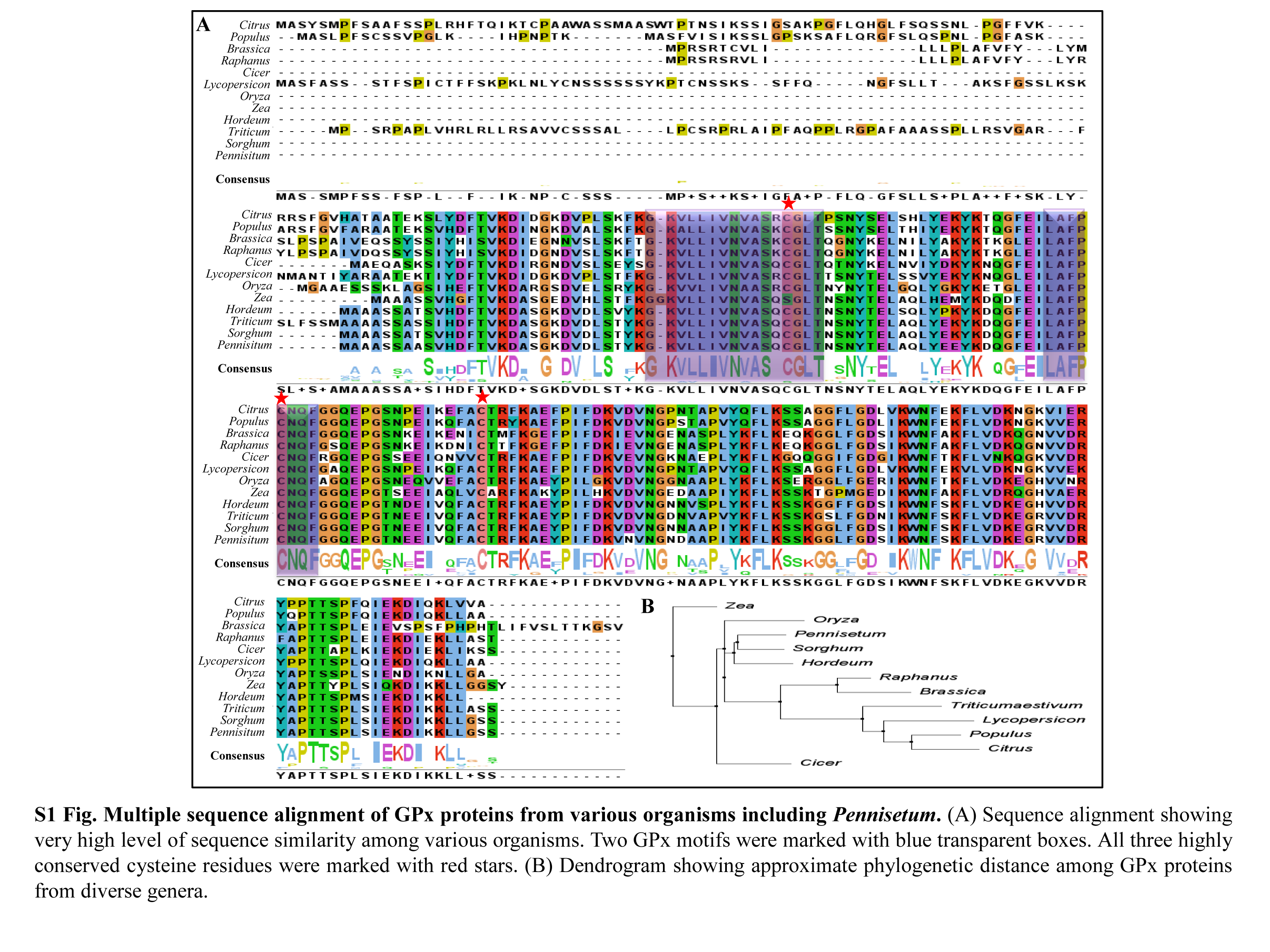

Supplement: S1 Fig — (TIF) [file pone.0143344.s001.tif]

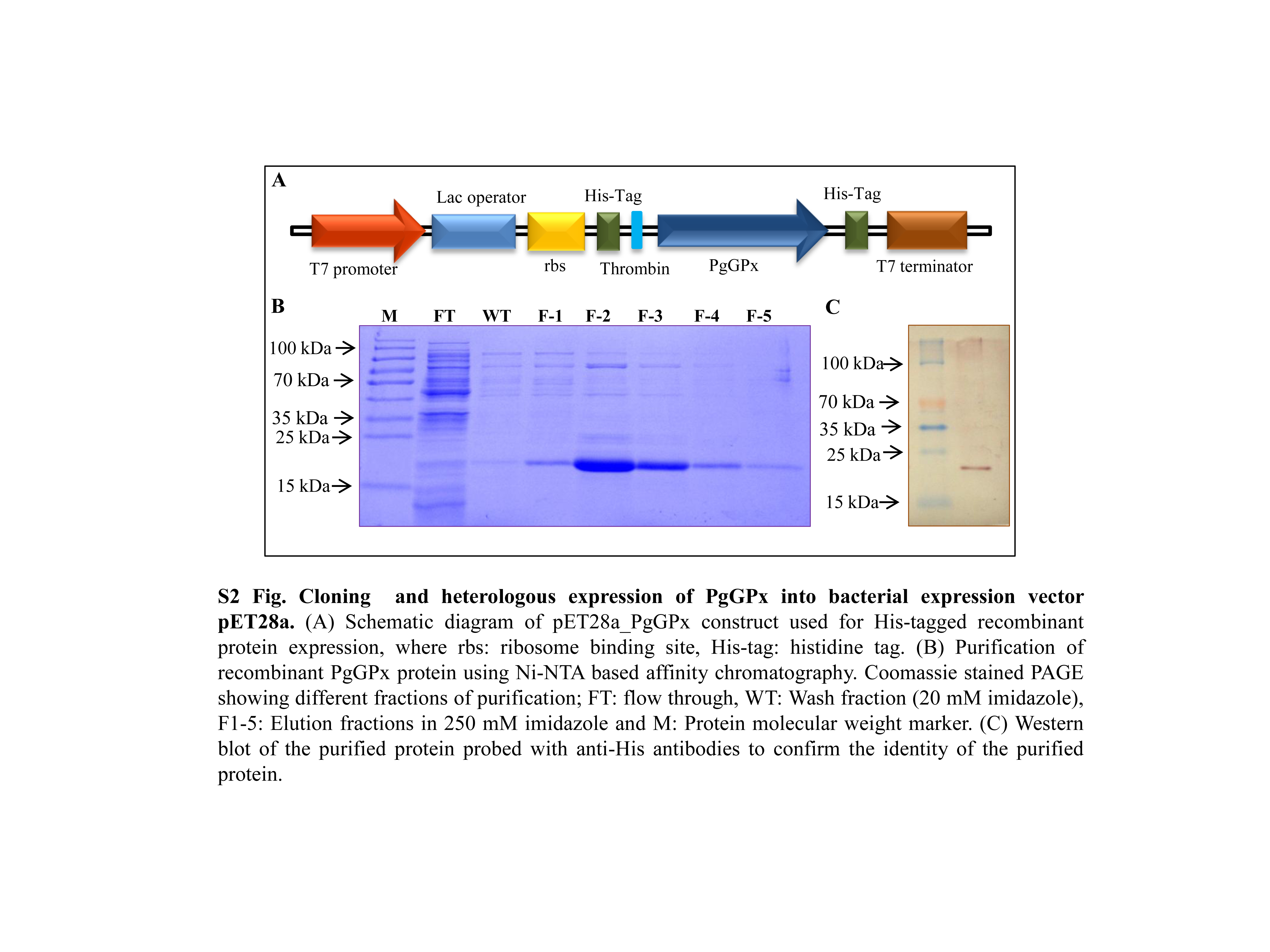

Supplement: S2 Fig — (TIFF) [file pone.0143344.s002.tiff]

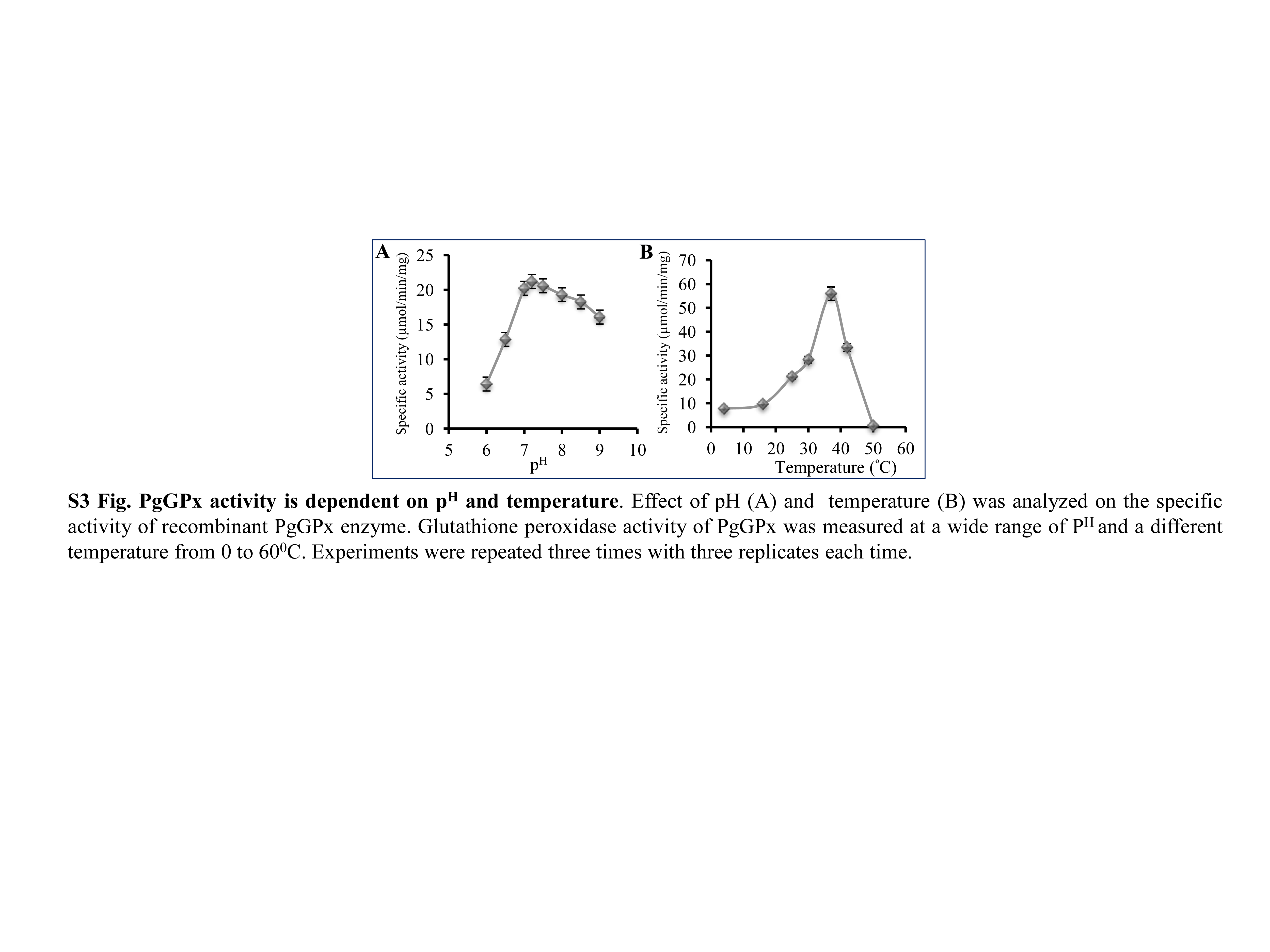

Supplement: S3 Fig — (TIFF) [file pone.0143344.s003.tiff]

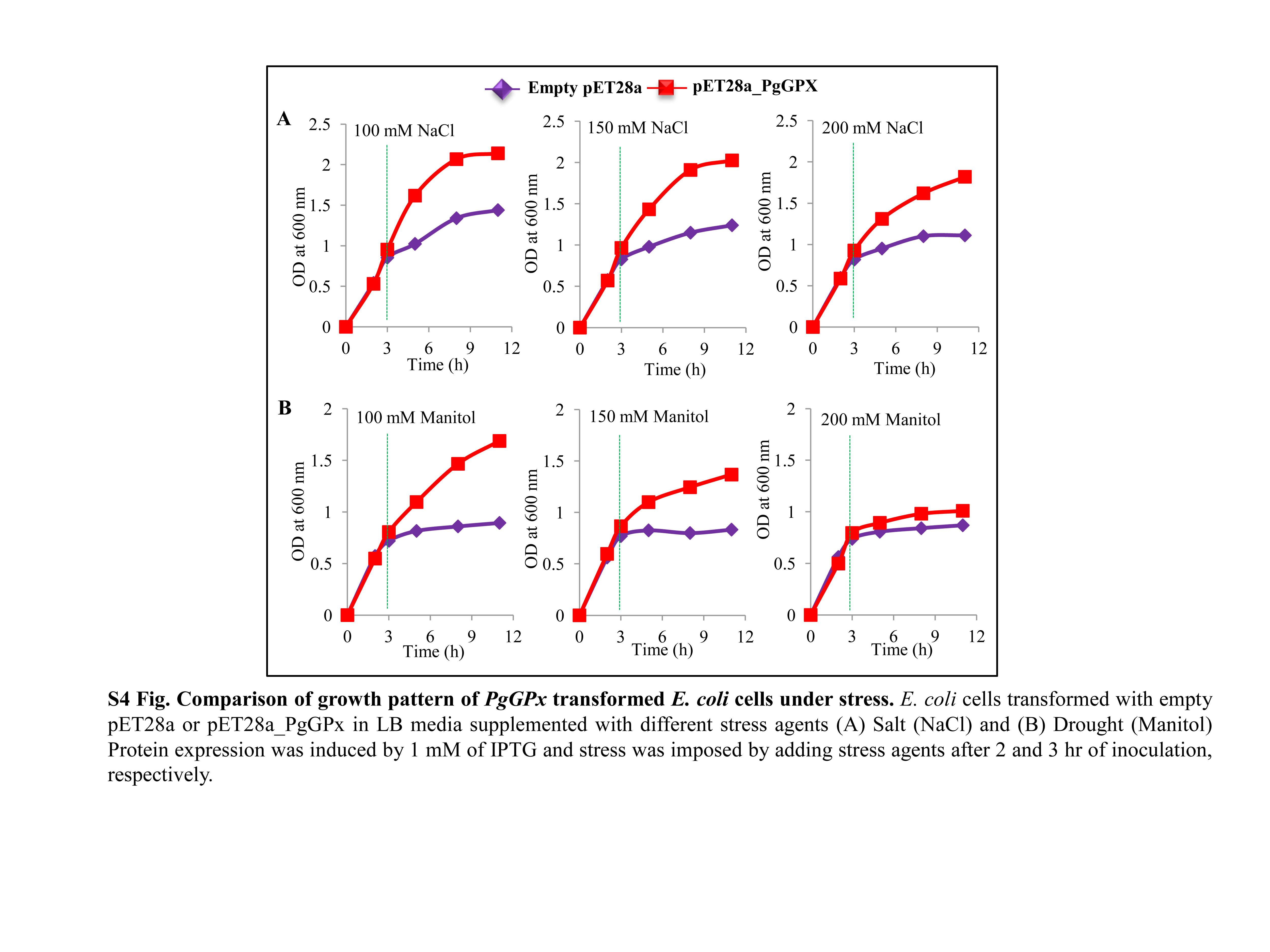

Supplement: S4 Fig — (TIFF) [file pone.0143344.s004.tiff]
