# Supplementary material for: Glutathione Peroxidase of Pennisetum glaucum (PgGPx) Is a Functional Cd2+ Dependent Peroxiredoxin that Enhances Tolerance against Salinity and Drought Stress
Source: PLoS One. 2015 Nov 23;10(11):e0143344. doi: 10.1371/journal.pone.0143344 (PMC4658160; doi:10.1371/journal.pone.0143344)
Supplement: S1 Table — (PDF) [file pone.0143344.s005.pdf]

S1 Table. Primers used in the study

Sl. No.	Primer name	Sequence (5'-3')
**1**	GPX NcoI F	TGGCCATGGCTGCTGCTTCGTCCGCC
**2**	GPX NotI R	AGCGGCCGCTTAAGAACTTCCGAGTAGTTTCT
**3**	GPX int_For	ATTGTCAACGTCGCATCCCA
**4**	GPX int _Rev	GGGCATAGCGATCCACAACA
**5**	GPX internal RT _For	TCAAGGCTGAGTACCCCATC
**6**	GPX internal RT _Rev	CTTGATGCTGTCACCGAAGA
**7**	GPX screen _For	GGCAAATAGTCTCCACCCCC
**8**	GPX screen _Rev	GGTTGCAAGGAAAAGCCAGG
**9**	Real_PgGPX_FOR	TATTGTCAACGTCGCATCCC
**10**	Real_PgGPX_REV	ACCTTGGTCCTTGTACTCCT
**11**	PgGPX RT_For	ACCTTGTAAACTGGAGCCGC
**12**	PgGPX RT_For	CATTGCATTGAACCTGATGC
**13**	hptII_F	ATGAAAAAGCCTGAACTCAC
**14**	hptII_R	CTATTTCTTTGCCCTCGGAC
**15**	Pg tubulin_For	GTGCTCTGAATGTGGATGTGAATG
**16**	Pg tubulin_Rev	ACCAACCTCCTCATAGTCCTTCTC
**17**	C42S_For	CATCCCAGTCTGGATTAACTA
**18**	C42S_Rev	CGACGTTGACAATAAGGAGA
**19**	C71S_For	CTTTTCCTTCCAACCAGTTTG
**20**	C71S_Rev	CCAGGATCTCAAAACCTTGG
**21**	C90S_For	AGTTTGCTTCCACACGCTTCA
**22**	C90S_Rev	GCACAATCTCCTCGTTTGTG
