# Supplementary material for: Glutathione Peroxidase of Pennisetum glaucum (PgGPx) Is a Functional Cd2+ Dependent Peroxiredoxin that Enhances Tolerance against Salinity and Drought Stress
Source: PLoS One. 2015 Nov 23;10(11):e0143344. doi: 10.1371/journal.pone.0143344 (PMC4658160; doi:10.1371/journal.pone.0143344)
Supplement: S2 Table — (PDF) [file pone.0143344.s006.pdf]

S2 Table. **Transformation efficiency of japonica rice cultivars.** Table showing the transformation efficiency *Agrobacterium* (LBA4404) mediated japonica rice cultivar with three replicates.

**No. of replicates**	**No. of seeds inoculated**	**No. of embryogenic calli generated**	**No. of calli used for transformation**	**No. of plantlets regenerated roots**	**No. of PCR positive plants**	**Transformation Efficiency (%) [a]**
1	100	85	50	21	9	
2	100	82	50	19	5	14%
3	100	89	50	24	7	

[a]Transformation efficiency (%) = No. of PCR positive plants /No. of calli inoculated with *Agrobacterium* X 100%
